# Orientation of Antigen Display on Self-Assembling Protein Nanoparticles Influences Immunogenicity

**DOI:** 10.3390/vaccines9020103

**Published:** 2021-01-29

**Authors:** Cosette G. Schneider, Justin A. Taylor, Michael Q. Sibilo, Kazutoyo Miura, Katherine L. Mallory, Christopher Mann, Christopher Karch, Zoltan Beck, Gary R. Matyas, Carole A. Long, Elke Bergmann-Leitner, Peter Burkhard, Evelina Angov

**Affiliations:** 1 Malaria Biologics Branch, Walter Reed Army Institute of Research, Silver Spring, MD 20910, USA; cosette.g.schneider.ctr@mail.mil (C.G.S.); justin.a.taylor62.ctr@mail.mil (J.A.T.); michael.q.sibilo.ctr@mail.mil (M.Q.S.); ktmallory@gmail.com (K.L.M.); cmann98@gmail.com (C.M.); elke.s.bergmann-leitner.civ@mail.mil (E.B.-L.); 2 Oak Ridge Institute for Science and Education, Oak Ridge, TN 37831, USA; 3 Parsons Corporation, Centreville, VA 20120, USA; 4 Laboratory of Malaria and Vector Research, National Institute of Allergy and Infectious Diseases, Rockville, MD 20892, USA; kmiura@niaid.nih.gov (K.M.); clong@niaid.nih.gov (C.A.L.); 5 Laboratory of Antigen and Adjuvants, US Military HIV Research Program, Walter Reed Army Institute of Research, Silver Spring, MD 20910, USA; christopher.p.karch.ctr@mail.mil (C.K.); zoltan.beck@gmail.com (Z.B.); gary.r.matyas.civ@mail.mil (G.R.M.); 6 Henry Jackson Foundation, Bethesda, MD 20817, USA; 7 Alpha-O Peptides AG, 4125 Riehen, Switzerland; peter.burkhard@aopeptides.ch

**Keywords:** self-assembling protein nanoparticles, PfCelTOS, PfMSP1_19_, vaccine, multi-stage, display

## Abstract

Self-assembling protein nanoparticles (SAPN) serve as a repetitive antigen delivery platform with high-density epitope display; however, antigen characteristics such as size and epitope presentation can influence the immunogenicity of the assembled particle and are aspects to consider for a rationally designed effective vaccine. Here, we characterize the folding and immunogenicity of heterogeneous antigen display by integrating (a) dual-stage antigen SAPN presenting the *P. falciparum* (*Pf*) merozoite surface protein 1 subunit, PfMSP1_19_, and *Pf* cell-traversal protein for ookinetes and sporozoites, PfCelTOS, in addition to (b) a homogenous antigen SAPN displaying two copies of PfCelTOS. Mice and rabbits were utilized to evaluate antigen-specific humoral and cellular induction as well as functional antibodies via growth inhibition of the blood-stage parasite. We demonstrate that antigen orientation and folding influence the elicited immune response, and when appropriately designed, SAPN can serve as an adaptable platform for an effective multi-antigen display.

## 1. Introduction

In 2019, the World Health Organization estimated 229 million cases of malaria and roughly 409,000 deaths worldwide, underscoring its continued prevalence as a global health threat [1]. In high-transmission areas, children under the age of five are among the most vulnerable, accounting for 67% of global malaria deaths [1]. Malaria infection occurs when infected *Anopheles* mosquitoes introduce *Plasmodium* parasites into the host during a blood meal. The introduced sporozoites invade hepatocytes, and subsequent blood-stage parasitemia leads to the symptoms of malaria. Notably, *Plasmodium falciparum (Pf)* is the leading cause of malaria morbidity and mortality, and in 2017, 2.6 billion people inhabited areas at risk of *P. falciparum* transmission, prompting this parasite species to be the focus of extensive efforts for vaccine development [2].

Although antimalarial drugs, exposure-reduction through use of bed nets, and vector control have reduced the incidence of malaria [3,4,5], to date, there is no licensed, efficacious malaria vaccine to support effective control and elimination of disease. The most advanced candidates, RTS,S and PfSPZ vaccines, while attaining high levels of protection against homologous strain parasites in controlled human malaria infections [6,7], achieved partial efficacy against natural infection [7,8,9,10,11,12,13,14]. The lack of effective vaccines and the severity of malaria infections necessitate the evaluation of novel vaccine technologies.

Given that *P. falciparum* displays highly variable antigens and shifts its dominant antigen expression throughout its life stages, a broadly effective vaccine will require a combination of antigens targeting discrete stages of development. The strategy described here combines a pre-erythrocytic and a blood-stage target to increase the breadth of immune responses. The latter, the major merozoite surface protein 1 (PfMSP1), is an extensively studied erythrocytic-stage parasite antigen thought to play a role in the invasion of red blood cells [15] and the focus of malaria blood-stage vaccine development [16,17,18]. PfMSP1 undergoes two essential, successive proteolytic events, resulting in the cleavage of the C-terminal 42 kDa fragment (PfMSP1_42_) into a 33 kDa (PfMSP1_33_) and a 19 kDa fragment (PfMSP1_19_) [19,20]. The latter remains GPI-anchored to the merozoite surface during invasion [19,20,21,22] and comprises two structured epidermal growth factor-like (EGF) domains [19,23], each containing three disulfide bridges [23] that assemble to form well-defined B cell epitopes [24,25]. Studies in endemic populations have supported the role of antibodies targeted to the C-terminus of PfMSP1 in a protective response [26,27,28]. Vaccine efforts to PfMSP1 have focused on the C-terminal portion, and several clinical studies have been undertaken to evaluate recombinant protein vaccines based on PfMSP1 fragments, including PfMSP1_19_ [29] and PfMSP1_42_ [30,31,32]. The latter vaccine, based on a PfMSP1_42_ 3D7 allele, FMP1/AS02A, advanced to a pediatric Phase 2b trial in Western Kenya, and while the vaccine elicited high antibody titers, it failed to reduce parasitemia or incidence of clinical malaria against the predominant circulating parasite strains [33]. Nominally, of the recombinant protein approaches, none elicited high levels of functional antibodies against parasites in in vitro growth inhibition assays (GIA) [29]. Therefore, strategies leading to improvements in antigen expression, presentation, and delivery are the focus for this target. Alternately, the cell-traversal protein for ookinetes and sporozoites (PfCelTOS) is a conserved pre-erythrocytic-stage antigen, having a role in traversal of both the mosquito and vertebrate host cells [34]. Pre-clinical studies of recombinant PfCelTOS demonstrated that vaccines based on this target induce functional immunity against the parasite developmental stages [35,36,37,38,39]. A vaccine against PfCelTOS offers the possibility to halt development during both mosquito and liver stages, respectively [34,40].

Recently, particulate delivery systems such as protein nanoparticles have emerged as promising platforms for vaccine development, overcoming the potentially limited immunogenicity of soluble subunit vaccines [41,42,43,44,45,46,47,48,49,50]. One particularly encouraging particulate delivery system is the self-assembling protein nanoparticle (SAPN), a versatile vaccine platform for multi-epitope display. SAPN form through the oligomerization of N-terminal pentameric and C-terminal trimeric coiled-coil sequence elements [51,52]. As the protein monomers oligomerize, the densely packed surface antigens present across icosahedral symmetry, forming a roughly spherical nanoparticle. This packed and ordered array of epitopes mimics that of strongly immunogenic virus capsids [53], while promoting highly specific immune responses similar to those elicited by peptide-based vaccines. In contrast to soluble proteins, the organized array of antigens on these particle surfaces are configured for T-cell independent, B-cell activation, by stimulating B-cell receptors to induce proliferation, thereby improving the immunogenicity of poorly immunogenic antigens [54,55]. Furthermore, SAPN offer the possibility to customize display on both the N- and C-termini to gain a more native-like, unconstrained structure, allowing conformational epitopes to form. In the same way, linear T-cell epitopes can be incorporated in the coiled-coil core sequence to activate both cellular and humoral immune responses [56]. In contrast to multiple antigenic peptides (MAP) [57,58], the production of SAPN vaccines is relatively robust, efficient, and cost-reductive. To date, SAPN have been engineered to display epitopes for malaria [59], influenza [60,61], HIV [62,63], SARS [64], and toxoplasmosis [65].

Motivated by the promise of SAPN’s flexible-by-design nature, we sought to investigate the influence of antigen orientation on elicited immune responses in mice and rabbits. SAPN co-displaying PfCelTOS and PfMSP1_19_ were expressed from both the N- and C-termini on monomers in alternate configurations (^red^PfMSP1_19_-PfCelTOS, ^ox^PfMSP1_19_-PfCelTOS, and PfCelTOS-^ox^PfMSP1_19_). The constructs were purified under either non-denaturing, oxidative-purification conditions (^ox^PfMSP1_19_) or denaturing, reductive conditions, (^red^PfMSP1_19_) followed by a four-step refolding process. In addition, a SAPN displaying a copy of PfCelTOS on each-termini was developed to determine whether a homogeneous molecule was superior to the chimeras that included PfMSP1_19_, addressing the possibility of immune competition, interference, or diversion. BALB/c mice were immunized with SAPN in adjuvant, either AddaVax™, an MF-59-like squalene-based oil-in-water emulsion, or Army Liposomal Formulation (ALF) containing a synthetic form of monophosphoryl lipid A (3D-PHAD) and the detoxified derivative QS-21 (ALFQ). Adjuvants with similar immunostimulatory molecules have been extensively used in human studies [66] and have been shown to enhance specific immune responses to antigen targets [67]. The impact of antigen localization to the N- or C-terminus on epitope accessibility was assessed by dot blotting using positional and conformational monoclonal antibodies. Humoral and cellular responses were assessed by antigen-specific ELISAs, parasite growth inhibition assays (GIA), indirect immunofluorescence assays (IFA), and by ELISpot and MSD, to assess cellular cytokine profiles, respectively, in mice and rabbits. In mice, humoral and cellular responses indicated that antigen conformation and localization on SAPN played a significant role on host immune responses. In rabbits, the growth inhibition assay (GIA) was performed to evaluate the ability of antibodies to limit blood-stage parasite growth in vitro. Thus, the simultaneous display of an erythrocytic antigen, PfMSP1, and a pre-erythrocytic antigen, PfCelTOS, on SAPNs potentially overcomes the requirement for ad hoc combinations of two antigens, i.e., cocktail, in a vaccine approach.

## 2. Materials and Methods

### 2.1. SAPN Design and Molecular Clones

**Figure 1:**
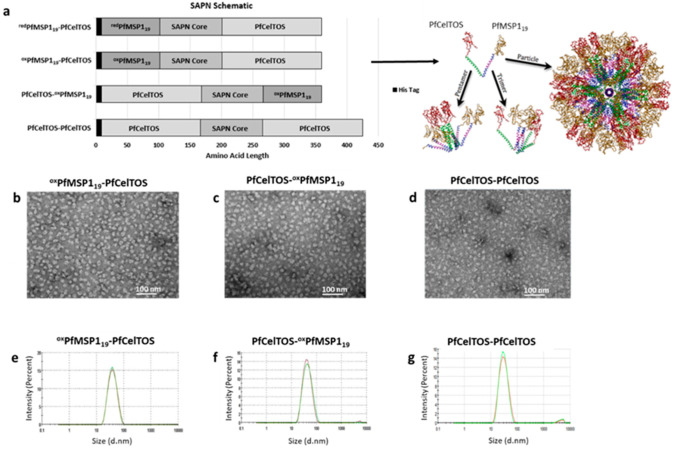
Self-assembling protein nanoparticles’ (SAPN) composition and particle quality. (**a**) Schematic diagram of SAPN with a theoretical model of PfCelTOS-^ox^PfMSP1_19_ monomers assuming icosahedral symmetry. (**b**–**d**) TEM of ^ox^PfMSP1_19_-PfCelTOS (**b**), PfCelTOS-^ox^PfMSP1_19_ (**c**), and PfCelTOS-PfCelTOS (**d**). (**e**–**g**) Hydrodynamic diameter size distribution of ^ox^PfMSP1_19_-PfCelTOS (**e**), PfCelTOS-^ox^PfMSP1_19_ (**f**), and PfCelTOS-PfCelTOS (**g**) determined by dynamic light scattering (DLS).

Malaria-specific sequences integrated into monomer polypeptides of SAPN proteins were derived from *P. falciparum* MSP1_19_ Vietnam Oak-Knoll (FVO) strain (accession # L20092.1) or *P. falciparum* CelTOS 3D7 strain (accession # AB194052.1) [35]. Malaria genes were codon harmonized for optimal expression in *E. coli* [68]. Coding sequences were synthesized by ATUM (DNA2.0, Newark, CA, USA) and cloned into a modified pET (K-) expression vector (Novagen, Madison, WI, USA). Full-length PfCelTOS comprises amino acids 25–182 from the native sequence. Recombinant subunit fragments of N-terminal and C-terminal PfCelTOS comprise amino acids 25–148 and 83–182, respectively. All recombinant clones included a 16 amino acid N-terminal histidine tag and linker sequence (Figure 1a). Details of the SAPN amino acid sequences are provided in Supplementary Figure S1.

### 2.2. Expression in BL21 [DE3] E. coli

BL21 [DE3] *E. coli* was transformed by electroporation using BioRad^®^ Gene Pulser Xcell™ according to standard protocol. Protein expression was performed using terrific broth (TB) media and 40 µg/mL kanamycin and expression induced by addition of 0.1 mM IPTG at mid-log growth phase. Expression levels were monitored by SDS PAGE/Coomassie blue staining (data not shown). 

### 2.3. Protein Purification

#### 2.3.1. Ni-NTA

Briefly, cells were resuspended in 12 volumes of Lysis buffer per gram of cell paste (*w/v*) and homogenized using an Ultra Turrax™ homogenizer (all buffer conditions are described in Supplementary Table S1). The cell suspension was mircofluidized (M110Y Microfluidizer^®^) and the final buffer adjusted to match the Ni^+2^-NTA Equilibration buffer. The lysate was lightly stirred at room temperature before centrifugation at 13,000 rpm (4 °C, 30 min). Supernatant was applied onto a gravity flow column followed by 15 column volumes (cv) of Equilibration buffer and 10 cv of each Wash buffer. The protein was eluted with 5cv Elution buffer and dialyzed using 12–14,000 molecular weight cut-off (MWCO) regenerated cellulose membrane (Spectrum Spectra/Por, New Brunswick, NJ) overnight at 4 °C in either Q-Sepharose Equilibration or SP-Sepharose Equilibration buffer depending on the product.

#### 2.3.2. SP-Sepharose

An SP-Sepharose column (GE Healthcare, Chicago, IL, USA) was equilibrated with 10cv SP-Equilibration buffer (4 °C, overnight). Dialysate was applied to the column, washed with 10cv SP Equilibration buffer, 10cv SP-Wash buffer, and eluted with 7cv SP-Elution buffer (Supplementary Table S1). The elution fraction was dialyzed in a 12–14,000 MWCO membrane in Q-Equilibration buffer (4 °C, overnight).

#### 2.3.3. Q-Sepharose

A Q-Sepharose column (GE Healthcare, Chicago, IL, USA) was equilibrated with 10 cv Q-Equilibration buffer before applying the dialyzed Ni-NTA or SP-Sepharose elution. The column was washed with 10 cv Q-Equilibration buffer, 10 cv Q-Wash buffer, and eluted with 5 cv Q-Elution buffer (Supplementary Table S1). The elution fraction was dialyzed using a 12–14,000 MWCO membrane in Refolding buffer 1 (4 °C, overnight).

### 2.4. Stepwise Refolding

To allow for the proper assembly of SAPN particles, the purified proteins were dialyzed using a 12–14,000 MWCO membrane at 2 h intervals at room temperature, in refolding buffers with decreasing concentrations of urea until the final buffer conditions were achieved (Supplementary Table S2). To ensure homogeneity, the final samples were centrifuged at 14,000 rpm and then filtered through either a 0.1 or 0.2 µm syringe filter. Bacterial endotoxin content was determined using the Limulus Amebocyte Lysate (LAL) Endotoxin Analysis Kit (Associates of Cape Cod Inc., E. Falmouth, MA, USA).

### 2.5. Dynamic Light Scattering (DLS)

SAPN were analyzed by dynamic light scattering (DLS) using a Malvern Zetasizer Nano S (Malvern, Worcestershire, UK) with a 633 nm laser and a ZEN2112 High Precision Cell quartz cuvette (Malvern, Worcestershire, UK). Particle size distributions were determined through the measurement of the hydrodynamic diameter at 25 °C. Each sample was run twice, and the average reading was recorded. Particle size and the polydispersity index was calculated using Malvern Panalytical Software. Polydispersity is calculated using the following formula $PDI=M_{w}/M_{n}$, where $M_{w}$ is the weight average molar mass, and $M_{n}$ is number average molar mass [69].

### 2.6. Transmission Electron Microscopy (TEM)

SAPN were adsorbed onto carbon-coated grids (Electron Microscopy Sciences Inc., Hatfield, PA, USA), washed twice with double distilled water, and negatively stained with 2% uranyl acetate (Electron Microscopy Sciences Inc., Hatfield, PA, USA). Electron micrographs were taken on a Hitachi HT7700 Transmission Electron Microscope with a LaB6 electron gun.

### 2.7. Dot Blot

Two hundred and fifty nanograms of each protein were spotted on a nitrocellulose membrane (Invitrogen, Carlsbad, CA, USA) and blocked in Milk Powder Solution (1X PBS, 0.1% Tween 20, 0.5 g/10mL milk powder) for 30 min at room temperature. Membranes were incubated with the appropriate primary antibodies, diluted in 1X PBS with 0.1% Tween 20, for 1 h at room temperature. Rabbit polyclonal antibodies (anti-PfCelTOS and anti-PfMSP1_42_) were diluted at 1:20,000, and monoclonal antibodies (3D11.D4, 4H9.C3, 3C3.B3, 5.2, 12.10, 1E1, and 2.2) were diluted at 1:10,000. Membranes were washed three times at five-minute intervals with 1XPBS with 0.1% Tween 20. Membranes were incubated with the appropriate secondary antibodies, diluted in 1X PBS with 0.1% Tween 20 for 1 h at room temperature. Membranes incubated with a polyclonal primary antibody were incubated with goat anti-rabbit IgG (AP) secondary antibody (1:10,000 [Invitrogen, Carlsbad, CA, USA]), and membranes incubated with a monoclonal primary antibody were incubated with goat anti-mouse IgG (H + L) AP secondary antibody (1:5,000 [Invitrogen, Carlsbad, CA, USA]). Membranes were washed as described above. Membranes were developed in alkaline phosphatase (AP) buffer (5M NaCl, 1M MgCl_2_, 1M Tris-HCl, pH 9.5) with 5-bromo-4-chloro-3′-indolyphosphate/nitro-blue tetrazolium (BCIP/NBT) (Sigma-Aldrich, St. Louis, MO, USA) for 15 min, or until development was complete. Development was arrested by washing with UltraPure Water, and membranes were air-dried between filter paper overnight.

### 2.8. Immunization

Female, BALB/c mice (The Jackson Laboratory, Bar Harbor, ME, USA) were immunized with 0.3, 1, 3, 10, or 30 µg of SAPN, 10 µg of recombinant protein rPfCelTOS, or 5 µg of recombinant protein rPfMSP1_42_ in the thigh muscle (50 µL in each thigh) three times at three-week intervals. All SAPN and recombinant proteins were formulated with AddaVax™ adjuvant (squalene-based oil-in-water emulsion [Invivogen, San Diego, CA, USA]), except for the PfCelTOS-PfCelTOS SAPN, which was formulated with and without Army Liposome Formulation with QS-21 (ALFQ) adjuvant. Mice were bled from the tail vein the day before each immunization and two weeks after the third immunization, at which time they were exsanguinated, and the corresponding sera were stored at −80 °C. All mouse procedures were conducted per the Institutional Animal Care and Use Committee (IACUC) at Walter Reed Army Institute of Research, Silver Spring, MD, USA.

Female, New Zealand white rabbits were immunized via intramuscular route three times at three-week intervals with either 0.5, 5, 15, or 50 µg PfCelTOS-PfMSP_19_, 50 µg PfMSP_19_-PfCelTOS or 50 µg rPfMSP1_42_ FVO in AddaVax™. Rabbits were exsanguinated two weeks following the last immunization. Noble Life Sciences Inc. performed rabbit studies, under AAALAC accreditation, OLAW assurance, USDA license, and efficient IACUC review processes.

### 2.9. Antibody Concentration ELISA

Ninety-six-well 2HB Immulon plates (Thermo Scientific, Waltham, MA, USA) were coated with 25 ng/well of rPfCelTOS, 15 ng/well of N-terminal PfCelTOS, 15 ng/well of C-Terminal PfCelTOS, or 35 ng/well of rPfMSP1_42_ in 1XPBS, pH 7.4 (Quality Biological, Gaithersburg, MD). An IgG standard plate run in parallel with the test plates was coated with two-fold serial dilutions of mouse IgG (1 mg/mL [Invitrogen, Waltham, MA, USA]) in 1XPBS, pH 7.4, starting with a 1:1000 dilution, then applied in triplicate on a 96-well plate. All plates were incubated overnight at 4 °C in a humidity chamber. Following the incubation, wells were blocked with 1X PBS/1% BSA (VWR, Chicago, IL, USA) for 1 h at 22 °C in a humidity chamber. Individual sera were diluted in 1X PBS/1% BSA to appropriate dilutions, then applied in triplicate to the test plate. Known concentrations of antibody to rPfCelTOS were applied in triplicates to the test plate as a positive control. 1X PBS/1% BSA was added to all wells of the standard plate, and all plates were incubated for 2 h at 37 °C. The secondary antibody (anti-mouse AP Conjugate [Promega, Madison, WI, USA]) was diluted in 1X PBS/1% BSA at 1:1000 and added to all wells, then incubated for 1 h at 22 °C in a humidity chamber. Wells were developed using the BluePhos Phosphatase Substrate 2-Component System (KPL, Milford, MA, USA) for 15 min at 22 °C. Development was arrested with 2X AP Stop Solution (SeraCare, Milford, MA, USA), then wells were detected using an M2 spectrophotometer (Molecular Devices, Downington, PA) at 630 nm. Between steps, wells were washed three times with wash buffer (1X PBS, 0.05% Tween 20, 0.1% chlorohexidine [*w/v*]).

### 2.10. Titration ELISA

Ninety-six-well 2HB Immulon plates (Thermo Scientific, Waltham, MA, USA) were coated with 35 ng/well of rPfMSP1_42_ antigens: FVO or 3D7, 25 ng/well of rPfCelTOS, 15 ng/well of N-terminal PfCelTOS, or 15 ng/well of C-Terminal PfCelTOS in 1X PBS, pH 7.4 and incubated overnight at 4 °C in a humidity chamber. Following the incubation, wells were blocked with 1X PBS/1% BSA (VWR, Chicago, IL, USA) for 1 h at 22 °C in a humidity chamber. Individual sera were diluted in 1X PBS/1% BSA, then serially diluted 2-fold down the plate and incubated for 2 h at 22 °C in a humidity chamber. The secondary antibody (HRP conjugated goat anti-mouse IgG [KPL, Milford, MA, USA] or AP-conjugated anti-rabbit IgG antibody [Promega, Madison, WI, USA]) was diluted in 1X PBS/1% BSA at 1:4000 and added to all wells, then incubated for 1 h at 22 °C in a humidity chamber. Wells were developed using the ABTS Peroxidase Substrate 2-Component System (for mouse [KPL, Milford, MA, USA]) or p-nitrophenyl phosphate (pNPP) tablets (Thermo Scientific, Rockford, IL, USA) dissolved in AP buffer (for rabbit [100 mM NaCl, 5 mM MgCl_2_, 100 mM Tris-HCl, pH 9.5]) for 1 h at 22 °C and detected using an M2 spectrophotometer (Molecular Devices, Downington, PA, USA) at 414 or 405 nm, respectively. Between steps, wells were washed three times with wash buffer (1X PBS, 0.05% Tween 20, 0.1% chlorohexidine [*w/v*]). Antibody titer is the dilution factor required to achieve an optical density of 1.0.

### 2.11. Growth Inhibition Assay (GIA)

Two weeks following the last immunization, rabbits were exsanguinated, and total IgG was purified using Protein G Sepharose. Growth inhibition assays were performed using purified IgGs. Briefly, each test IgG (5, 2.5 or 1.25 mg/mL in a final test well) was incubated with synchronized late trophozoite *P. falciparum* FVO (homologous) or 3D7 (heterologous) parasites for 44–48 h, and relative parasitemia levels were quantified by measuring the level of parasite lactate dehydrogenase.

### 2.12. Immunofluorescence Assay

Immunofluorescence assays (IFA) were performed as previously described [35] using salivary gland *P. falciparum* sporozoites. Spots were incubated with DAPI (1:50, Southern Biotech, Birmingham, AL, USA) in addition to goat anti-mouse IgG-FITC (1:100, Southern Biotech, Birmingham, AL, USA).

### 2.13. ELISpot

ELISpot was performed as previously described [35] and according to the manufacturer’s instructions (R&D Systems, SEL485 and SEL404, Minneapolis, MN, USA). Briefly, rPfCelTOS (10 µg/mL) was used as the stimulating antigen. Hamster anti-mouse CD3e (1 µg/mL [BD Biosciences, 553057, San Jose, CA, USA]) was used as a positive control for cell stimulation. The negative control was culture media in place of a stimulating antigen. Splenocytes were plated at 2 × 10^5^ cell/well. Plates were incubated for 48 h at 37 °C with 5% carbon dioxide. Spot counting was performed using an AID ELISpot Reader (Autoimmun Diagnostika, Strassberg, Germany).

### 2.14. Meso Scale Discovery Assay

Mouse splenocytes were plated at 400,000 cells/well in 96-well flat bottom plates (Costar 3595). Cells were stimulated with 10 µg/mL rPfCelTOS protein for 48 h at 37 °C with 5% carbon dioxide, after which cell culture supernatants were harvested. Pro-inflammatory cytokines were quantified using V-PLEX Proinflammatory Panel 1 Mouse Kit (Meso Scale Discovery, K15048D) according to the manufacturer’s instructions. Cytokine levels were normalized for background secretion using the levels detected with cells incubated in culture media alone.

### 2.15. Statistical Analysis

Statistical significance of mouse serological responses and cellular and fine specificity responses were evaluated using the Kruskal–Wallis test with Dunn’s multiple comparisons test and Wilcoxon matched-pairs signed rank test, respectively. Rabbit responses were evaluated using single comparisons to the rMSP1_42_ group using multiple *t* tests, and the Holm-Sidak method. Differences in PfMSP1_42_ antigen strain responses were compared using Mann–Whitney multiple *t* tests, where *p* < 0.05 indicates statistical significance (GraphPad Prism 6.0, La Jolla, CA, USA).

## 3. Results

### 3.1. SAPN Design and Particle Quality

The SAPN structures generated in this study aimed to explore the effect of orientation of antigen display on the immunogenicity of a multi-stage malaria vaccine candidate. Sequences for the MSP1_19_ protein fragment from the Vietnam Oak-Knoll (FVO) strain of *P. falciparum* (PfMSP1_19_) and the full-length CelTOS protein from the 3D7 strain of *P. falciparum* (PfCelTOS) (Supplementary Figure S1) were designed for display on either the N- or C-termini of SAPN monomers (Figure 1a). SAPN were purified by consecutive Ni-NTA affinity and ion-exchange chromatography followed by a four-step refolding dilution process to remove chaotropic and reductive agents (Supplementary Tables S1 and S2). SAPN displaying PfMSP1_19_ and PfCelTOS were generated by two purification processes, one allowing for disulfide bridges to form first under non-denaturing, oxidative affinity chromatography (^ox^PfMSP1_19_) followed by denaturing conditions on ion exchange and the second using denaturing/reductive conditions throughout the chromatography (^red^PfMSP1_19_), which allowed for disulfide bridges to form during the final refolding steps. A SAPN displaying PfCelTOS (PfCelTOS-PfCelTOS) on both the N- and C- termini was generated to evaluate the influence of a homogeneous, albeit alternative configuration, on CelTOS-specific immune responses (Figure 1a, bottom).

Prior to evaluating the immunogenicity of SAPN, particle quality was assessed by DLS and TEM. TEM demonstrated the formation of homogenous populations of particles, revealing nearly spherical assembled particles approximately 20 nm in diameter (Figure 1b–d). DLS analysis confirmed the homogeneity of the populations and indicated hydrodynamic diameters of ^ox^PfMSP1_19_-PfCelTOS, PfCelTOS-^ox^PfMSP1_19_, and PfCelTOS-PfCelTOS of 34, 38, and 29 nm, respectively (Figure 1e–g) [70]. Polydispersity indices (PdI) calculated by Malvern Panalytical Software ranged from 0.180 to 0.190, indicating low propensity for aggregation. Therefore, these results suggest that SAPN form into homogenous populations and their formation is not affected by antigen localization, at least for these targets. Importantly, the particle sizes estimated by these methods are consistent with previous SAPN [71] and are indicative of the coiled-coil structure forcing self-assembly of the monomers as predicted in the theoretical model (Figure 1a).

### 3.2. SAPN Immunoreactivity Using CelTOS and MSP1_19_ mAbs

**Figure 2:**
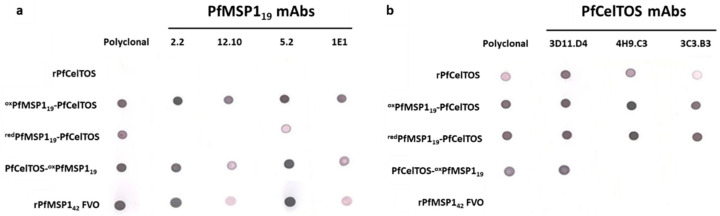
Antigen localization on SAPN influences immunoreactivity. (**a**,**b**) Reactivity of conformation-dependent *P. falciparum* MSP1_19_ (**a**) and *P. falciparum* CelTOS (**b**) monoclonal antibodies to the SAPN and recombinant proteins, *P. falciparum* MSP1_42_ and *P. falciparum* CelTOS. Nitrocellulose membranes were spotted with 250 ng of folded proteins and incubated with antibodies as described in Materials and Methods.

The immunoreactivity of SAPN was evaluated by using conformation-dependent/antigen-specific monoclonal antibodies. Immune recognition on SAPN is dependent on target epitope accessibility and the display of properly folded conformational epitopes. To characterize epitope accessibility and recognition, SAPN were spotted on nitrocellulose membranes and probed with antigen-specific polyclonal antibodies raised against either rPfCelTOS or rPfMSP1_42_ FVO and disulfide-dependent, conformation-specific PfMSP1_19_ and C-terminal PfCelTOS-specific mAbs. SAPN in which the disulfide bonds were formed in conjunction with the refolding step (^red^PfMSP1_19_-PfCelTOS) were not readily detected with PfMSP1_19_ mAbs, 2.2 and 12.10 [24], and 1E1 [20], while those oxidized prior to the refolding step (^ox^PfMSP1_19_-PfCelTOS) were recognized by all four PfMSP1_19_ mAbs (Figure 2a). ^red^PfMSP1_19_-PfCelTOS was weakly detected with mAb 5.2 suggesting some relevant structure formation (Figure 2a). Detection by PfCelTOS mAbs was not influenced by the purification conditions used for disulfide bridge formation on ^red^PfMSP1_19_-PfCelTOS versus ^ox^PfMSP1_19_-PfCelTOS, since it lacks disulfide structure and, in both cases, is localized to the C-terminus (Figure 2b). In contrast, PfCelTOS mAbs, either 4H9.C3 or 3C3.B8, did not recognize PfCelTOS on the N-terminus (PfCelTOS-^ox^PfMSP1_19_), possibly due to misfolding or epitope constrains. Only mAb 3D11.D4 recognized PfCelTOS oriented on the N-terminus of SAPN (Figure 2b). All PfMSP1 antibodies detected the recombinant, soluble PfMSP1_42_, but not rPfCelTOS, while, conversely, all PfCelTOS antibodies detected the recombinant, soluble PfCelTOS, but not the rPfMSP1_42_, indicating no cross-reactivity between these antigens (Figure 2a,b).

### 3.3. Epitope Density Influences Immunogenicity

**Figure 3:**
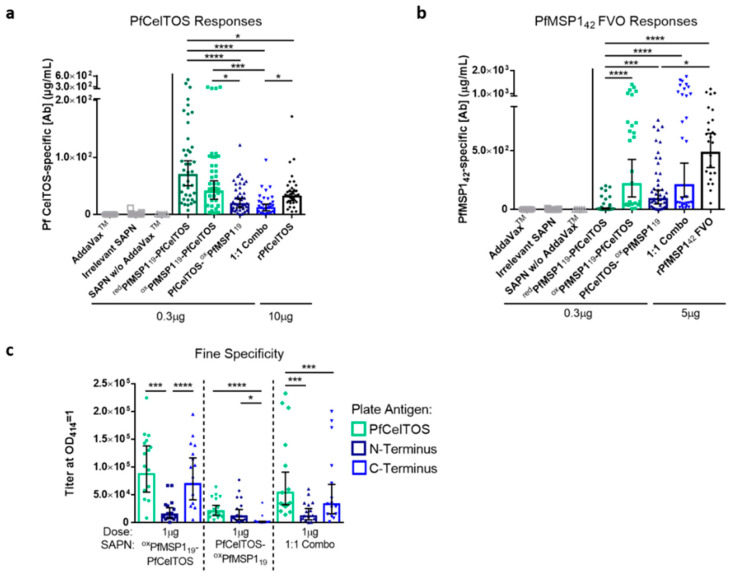
Antigen localization and epitope density on SAPN influences immunogenicity and antibody fine specificity. (**a**–**c**) BALB/c mice were immunized I.M. 3 times at a 3-week interval with ^red^PfMSP1_19_-PfCelTOS ((**a**,**b**), n = 39), ^ox^PfMSP1_19_-PfCelTOS ((**a**), n = 40; (**b**), n = 25; (**c**), n = 15), PfCelTOS-^ox^PfMSP1_19_ ((**a**,**b**), n = 38; (**c**), n = 15), or 1:1 Combo ((**a**,**b**), n = 30; (**c**), n = 15) SAPN (0.3 µg for (**a**,**b**) and 1 µg for (**c**)), recombinant *P. falciparum* CelTOS (10 µg, n = 35), or recombinant *P. falciparum* MSP1_42_ (5 µg, n = 25) in AddaVax™. The 1:1 Combo is an assembled 1:1 admixture of ^ox^PfMSP1_19_-PfCelTOS and PfCelTOS-^ox^PfMSP1_19_. Negative controls include immunization with AddaVax™ alone ((**a**,**b**), n = 25), irrelevant SAPN (**a**,**b**), n = 25), and 0.3 µg ^red^PfMSP1_19_-PfCelTOS in PBS (denoted as SAPN w/o AddaVax™; (**a**,**b**), n = 5). Antigen-specific antibody concentrations against *P. falciparum* CelTOS (**a**) and *P. falciparum* MSP1_42_ (**b**) and antigen-specific IgG titers against *P. falciparum* CelTOS and its N- and C-terminal subunits (**c**) were quantified in sera 2 weeks post-final immunization by ELISA. Final titers are shown for individual mice, with the geometric mean and 95% confidence intervals (CI). *: *p* < 0.05; ***: *p* < 0.001; ****: *p* < 0.0001 by the Kruskal–Wallis test with Dunn’s multiple comparisons (**a**,**b**) and the Wilcoxon matched-pairs signed rank test (**c**) (GraphPad Prism 6.0). Results are a meta-analysis of five independent experiments. Dotted lines separate the results for each SAPN.

Coiled coil proteins/peptides are attractive units for de novo design of ordered assemblies forming polyhedral, ring structures, and planar and linear lattices. Formation of high-density, repetitive, and ordered displays allow for enhanced immunogenicity compared to that induced by soluble proteins [72]. Immunogenicity of the SAPN and soluble proteins, rPfCelTOS and rPfMSP1_42_, were tested in BALB/c mice. Mice were immunized three times at three-week intervals with 0.3 µg SAPN and 10 µg rPfCelTOS or 5 µg rPfMSP1_42_ formulated in AddaVax™. Vaccine dosing based on molar equivalents of PfCelTOS delivered on SAPN approximated a 73-fold lower dose relative to the soluble protein (PfMSP1_19_-PfCelTOS, at 7.58 pmoles; PfCelTOS, at 555.6 pmoles, respectively). Similarly, for PfMSP1_19_ displaying SAPN, the moles of target antigen approximated a 16-fold lower dose than for the rPfMSP1_42_ (7.58 pmoles and 119.1 pmoles, respectively). The doses selected for the recombinant proteins are based on previous studies and served as reference controls [35]. The SAPN doses were nominally selected to assess the potential for antigen dose sparing. Negative controls, AddaVax™ alone, irrelevant SAPN, and 0.3 µg ^red^PfMSP1_19_-PfCelTOS in PBS (Figure 3a,b, denoted as SAPN w/o AddaVax™) were included to explore background responses elicited by the adjuvant or the SAPN core.

Localization of PfCelTOS to the C-terminus of SAPN (^red^PfMSP1_19_-PfCelTOS and ^ox^PfMSP1_19_-PfCelTOS) yielded a statistically higher antibody concentration relative to its N-terminal display (PfCelTOS-^ox^PfMSP1_19_) (Figure 3a). To address the effect of an alternate packing density, a 1:1 admixture comprising both N-terminal and C-terminal localized PfCelTOS was co-assembled (Combo [^ox^PfMSP1_19_-PfCelTOS and PfCelTOS-^ox^PfMSP1_19_]). Interestingly, the Combo SAPN yielded lower PfCelTOS antibody concentrations relative to the C-terminal PfCelTOS SAPN, but similar PfCelTOS antibody concentrations relative to the N-terminal PfCelTOS SAPN (Figure 3a). Therefore, antibody responses to PfCelTOS were reduced by either localizing to the N-terminus or by halving the epitope density at the C-terminus in the 1:1 Combo SAPN.

In contrast, N-terminal PfMSP1_19_ (^ox^PfMSP1_19_-PfCelTOS) yielded significantly higher PfMSP1 antibodies compared to ^red^PfMSP1_19_-PfCelTOS (Figure 3b), which lacked some disulfide bridge structures (Figure 2a). Localizing ^ox^PfMSP1_19_ to the N-terminus (^ox^PfMSP1_19_-PfCelTOS) or the C-terminus (PfCelTOS-^ox^PfMSP1_19_) did not yield significantly different antibody concentrations; however, PfCelTOS- ^ox^PfMSP1_19_ elicited lower antibody concentrations relative to rPfMSP1_42_ (Figure 3b). Antibody concentrations elicited by ^ox^PfMSP1_19_-PfCelTOS did not significantly differ from the 1:1 Combo, nor rPfMSP1_42_. Relative to the SAPN configuration, the results for MSP1 suggest that disulfide bond structure was more essential to the elicited response, rather than the antigen localization or epitope density. Importantly, the negative controls (AddaVax™ alone, empty SAPN, and 0.3 µg ^red^PfMSP1_19_-PfCelTOS in PBS) elicited universally negligible responses, suggesting no notable background response, and highlighted the need for adjuvant to induce antibody production (Figure 3a,b). Together, these results reveal that, for PfCelTOS, display on the C-terminus of SAPN and, for PfMSP1_19_, disulfide-dependent structure defined the immunogenicity outcome.

### 3.4. Orientation of PfCelTOS on SAPN Influences Antibody Specificity

To assess for antibody fine specificity induced by SAPN, BALB/c mice were immunized with 1 µg of ^ox^PfMSP1_19_-PfCelTOS, PfCelTOS-^ox^PfMSP1_19_, or a 1:1 assembled admixture (“Combo”; DLS, Z-average 29.14 nm, particle data not shown) formulated in AddaVax™. Antibody titers against full-length PfCelTOS, and the subunit fragments, N-terminus and C-terminus of PfCelTOS, were evaluated to assess whether antibody specificities were influenced by localization on SAPN. Localization of PfCelTOS to the C-terminus of the SAPN (^ox^PfMSP1_19_-PfCelTOS) elicited a significantly higher antibody titer against the PfCelTOS C-terminal subunit relative to the PfCelTOS N-terminal subunit (Figure 3c). The 1:1 Combo SAPN yielded antibody titers that mirrored the specificities elicited by PfCelTOS localized to the C-terminus but at a lower magnitude overall. In contrast, localization of PfCelTOS to the N-terminus of the SAPN (PfCelTOS-^ox^PfMSP1_19_) significantly diminished the antibody responses against the C-terminal subunit. The responses to the N-terminal subunit of PfCelTOS were universally weak, as each SAPN configuration produced an equally low antibody titer to this fragment. These results suggest that the location of PfCelTOS on SAPN mainly influenced antibodies targeting to the C-terminus of the antigen.

### 3.5. SAPN Induces Antibodies that Inhibit Parasites In Vitro

**Figure 4:**
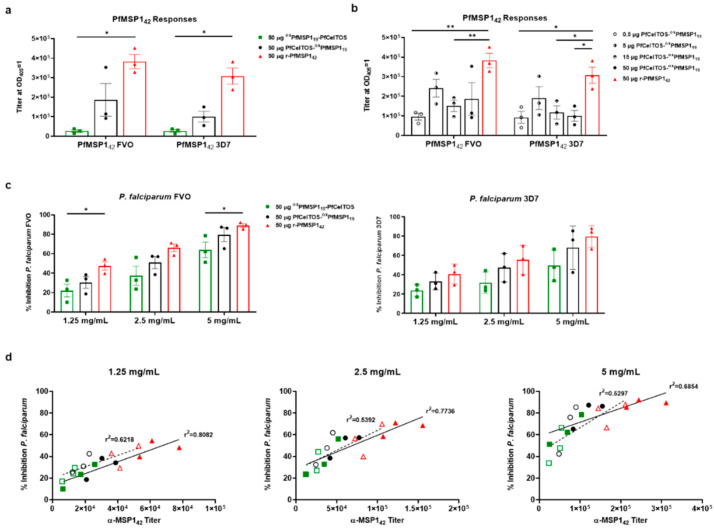
SAPN induces antibodies that inhibit parasites in vitro. Two weeks following the third I.M. immunization, rabbits were exsanguinated, and serum antibody titers were measured against recombinant proteins and blood-stage parasites. (**a**) Fifty micrograms dose response of PfCelTOS-^ox^PfMSP1_19_ (n = 3), ^ox^PfMSP1_19_-PfCelTOS (n = 3), and rPfMSP1_42_ (n = 3) to the Vietnam Oak-Knoll (FVO) and 3D7 PfMSP1_42_ antigens. (**b**) PfCelTOS-^ox^PfMSP1_19_ dose-response relationship compared to the previous 50 μg PfCelTOS-^ox^PfMSP1_19_ and rMSP1_42_ groups. (**c**) Total IgG was normalized to 1.25, 2.5, and 5 mg/mL. Fifty micrograms of each SAPN construct and rPfMSP1_42_ were tested for FVO and 3D7 parasite-specific growth inhibition by measuring the amount of parasite lactate dehydrogenase present. (**d**) Correlation graphs between purified IgG ELISA titer and percent inhibition at 1.25, 2.5, and 5 mg/mL with linear regression analysis. FVO strain titer and percent inhibition are represented by filled symbols and a solid trend line, while 3D7 strain titer and percent inhibition are represented by open symbols with a dash trend line. Note: there are overlapping symbols on the 2.5 mg/mL graph, where an ^ox^PfMSP1_19_-PfCelTOS sample has nearly identical titer (12,587, 11,760) and identical percent inhibition (23.6) for both FVO and 3D7 strains, respectively. Data are shown as the mean with +/− standard error of the mean. Statistical significance was determined using nonparametric multiple *t* test, the Holm–Sidak method comparing to the rPfMSP1_42_ standard group with *: *p* < 0.05; **: *p* < 0.01. Differences in PfMSP1_42_ strain responses were compared using Mann–Whitney tests.

Given the establishment of potent humoral immune responses in the murine model, the functionality of MSP1 antibodies against parasite growth in vitro was assessed in outbred, New Zealand white rabbits. Three rabbits per group were immunized with the respective immunogen formulated in AddaVax™ and sera were collected two weeks after the final immunizations. Ig titers were measured against rPfMSP1_42_ alleles, 3D7 and FVO (Figure 4a,b) and rPfCelTOS, in addition to PfCelTOS subunit fragments in order to deduce antibody fine specificities (Supplementary Figure S2a). Due to the immunodominant nature of the PfMSP1 response and the relatively lower PfCelTOS-specific titers, SAPN constructs and their dose-response relationships were only assessed for their MSP1 responses, and these were compared to a recombinant, soluble PfMSP1_42_ FVO (rPfMSP1_42_) protein, a vaccine candidate previously evaluated in the clinic [31].

The rPfMSP1_42_ protein stimulated higher overall antibody titers against both alleles of MSP1_42_ than either SAPN ^ox^PfMSP1_19_-PfCelTOS or PfCelTOS-^ox^PfMSP1_19_. Markedly, ^ox^PfMSP1_19_-PfCelTOS, when delivered at an equivalent dose, 50 µg (equal molar relative to MSP1_19_), with the N-terminal oriented MSP1_19_, induced significantly lower titers (Figure 4a). Conversely, for PfMSP1_19_ located on the C-terminal end of SAPN, antibodies were seven-fold higher compared to the inverse orientation. Interestingly, this difference was not observed in mice where antibody titers were not significantly impacted by orientation on SAPN for MSP1_19_ (Figure 4b). Cross-reactive antibody responses were not significantly different between the FVO and 3D7 plate antigens no matter the immunogen. These findings corroborated previous work with the FVO allele of PfMSP1 for inducing superior cross-reactive responses and was the basis for its selection in the current study [73]. Next, rabbits were vaccinated with PfCelTOS-^ox^PfMSP1_19_ in AddaVax™ at various antigen doses (0.5, 5, 15, and 50 µg) to deduce an optimal dose response curve. Antibody titers revealed a relative lack of responsiveness in the dose range tested (Figure 4b). Notably, this lack of dose responsiveness was paralleled for both alleles of rPfMSP1_42_, FVO and 3D7. While these findings may be intriguing, a larger sample size is warranted to fully assess SAPN dose response in rabbits, and therefore, the current findings may be more indicative of the small sample size.

To assess antibodies functionality against parasite growth, in vitro parasite growth inhibition assays (GIA) were performed using individual purified immunoglobulins from rabbits vaccinated with 50 µg of ^ox^PfMSP1_19_-PfCelTOS, PfCelTOS-^ox^PfMSP1_19_, or rPfMSP1_42_ formulated in AddaVax™. Protein G purified Igs were normalized to 1.25, 2.5, and 5 mg/mL and incubated with the blood stages of FVO or 3D7 parasites in vitro. Growth inhibition activity was quantified based on parasite lactate dehydrogenase detection [73]. At each concentration of Ig, inhibition trends were ^ox^PfMSP1_19_-PfCelTOS < PfCelTOS-^ox^PfMSP1_19_ < rPfMSP1_42_, against *P. falciparum* FVO parasites (Figure 4c). A similar trend was observed against *P. falciparum* 3D7 parasites. Generally, SAPN with PfMSP1_19_ localized on the N-terminus induced lower inhibition than in the inverse orientation (PfCelTOS-^ox^PfMSP1_19_), where there was no significant difference in inhibition detected compared to soluble rPfMSP1_42_, at any concentration of Ig. Similar to the antibody titers, there was no significant difference in parasite growth inhibition between the FVO and 3D7 strain parasites. Not surprisingly, in all cases, the purified Ig antibody titers (Supplementary Figure S2b) and percentage growth inhibition were directly correlated (r^2^ = 0.8082, 0.7736, 0.6854; 1.25, 2.5, 5 mg/mL, respectively, *P. falciparum* FVO responses) (Figure 4d: filled symbols with solid trend line); however, for the *P. falciparum* 3D7 responses, the r^2^ values were generally lower (r^2^ = 0.6218, 0.5392, 0.5297; 1.25, 2.5, 5 mg/mL, respectively, *P. falciparum* 3D7 responses) (Figure 4d: open symbols with dash trend line). For both sets of response curves, r^2^ values decreased as Ig concentrations increased, suggesting at higher antibody concentrations, responses reached a saturating limit. Thus, SAPN induced humoral responses to the conformation-dependent MSP1_19_, which were cross-reactive, functional, and compared favorably to the reference, rMSP1_42_.

### 3.6. Immunogenicity of Dual-Displaying PfCelTOS SAPN

**Figure 5:**
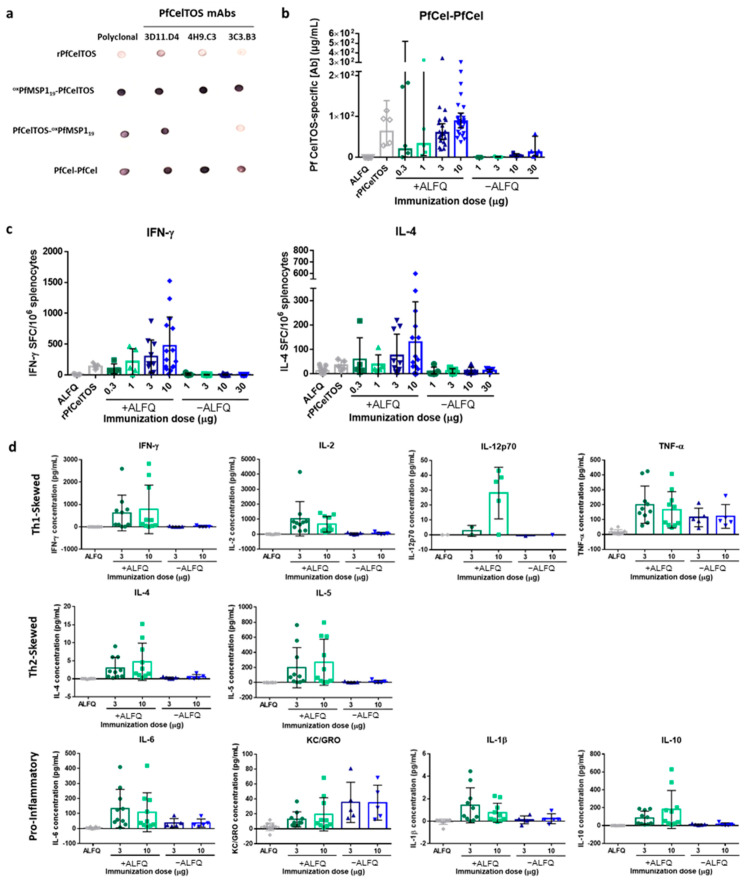
Immunogenicity of N-terminal and C-terminal displayed PfCelTOS on PfCel-PfCel SAPN. (**a**) Reactivity of *P. falciparum* CelTOS antibodies to PfCel-PfCel SAPN. Nitrocellulose membrane was spotted with 250 ng of folded proteins and incubated with antibodies as described in Materials and Methods. (**b**) BALB/c mice were immunized I.M. 3 times at a 3-week interval with PfCel-PfCel SAPN with (0.3 µg, n = 5; 1 µg, n = 5; 3 µg, n = 20; 10 µg, n = 25) and without (1 µg, n = 5; 3 µg, n = 5; 10 µg, n = 5; 30 µg, n = 5) Army Liposome Formulation with QS-21 (ALFQ) adjuvant, recombinant PfCelTOS (10 μg, n = 5) with ALFQ, or ALFQ alone (n = 20). Antigen-specific antibody concentrations against rPfCelTOS were quantified by ELISA. Final antibody concentrations are shown for individual mice, with the geometric mean and 95% CI. (**c**) IFN-γ- and IL-4-secreting cells were quantified by ELISpot after stimulation of splenocytes with rPfCelTOS. The number of cytokine-specific spot-forming cells (SFC) per 10^6^ splenocytes is shown for individual mice (n = 5 for all groups except the 3 µg and 10 µg doses with ALFQ and ALFQ alone groups, which have n = 10, n = 15, n = 15, respectively), with the mean and SD. (**d**) Cytokine production was quantified using the Meso Scale Discovery immunoassay. Cytokine-specific concentrations are shown for individual mice (n = 10 for groups with ALFQ, n = 5 for groups without ALFQ, except IL-12p70 for which some mice were below the level of detection), with the mean and SD. Results are representative of three independent experiments.

Despite the favorable immunogenicity of PfMSP1_19_ SAPN in rabbits, we sought to evaluate whether immune responses could be improved by increasing epitope density through the simultaneous display of a single target on both the N- and C-terminus of SAPN. A PfCelTOS-PfCelTOS SAPN (PfCel-PfCel) was produced and its immune recognition on dot blots and immunogenicity in mice was tested. To characterize the PfCel-PfCel SAPN relative to the single-antigen display SAPN, ^ox^PfMSP1_19_-PfCelTOS, PfCelTOS-^ox^PfMSP1_19_, and a soluble rPfCelTOS, 250 ng of protein was spotted onto nitrocellulose membranes and detected by PfCelTOS-specific polyclonal and monoclonal antibodies. C-terminal-specific mAbs detected epitopes on the C-terminally localized PfCelTOS SAPN (PfCel-PfCel and ^ox^PfMSP1_19_-PfCelTOS) at high densities relative to the recombinant, soluble protein; however, detection was significantly reduced when the PfCelTOS was localized to the N-terminus (PfCelTOS-^ox^PfMSP1_19_) for mAbs 4H9.C3 and 3C3.B3, but not mAb 3D11.D4, whose epitopes appear accessible in this configuration (Figure 5a). Not surprisingly, the dual displaying PfCelTOS SAPN (PfCel-PfCel) was recognized by all mAbs.

Next, we assessed the immunogenicity of PfCel-PfCel with increasing doses with and without potent adjuvant, ALFQ. ALFQ was selected for its’ reported balanced Th1/Th2 response compared to AddaVax™, which biases toward Th2 immunity. Notably, formulation with ALFQ yielded a significantly higher antibody concentration across all doses; whereas, in the absence of adjuvant, the responses were overall lower (Figure 5b). Increasing the SAPN dose did not yield significant differences in geometric mean antibody concentrations; however, the 95% confidence interval of the antibody responses narrowed with increasing doses. The range of antibody responses and the smaller confidence interval are likely due to the larger number of mice immunized with the higher doses than with lower doses. While the mean antibody concentrations elicited by SAPN varied, no differences were detected with responses induced by the rPfCelTOS. However, a trend was observed for higher doses leading to higher mean antibody concentrations. Importantly, antibodies raised to this SAPN were capable of recognizing native antigen on the *P. falciparum* sporozoites, by IFA (Supplementary Figure S3), suggesting display of some natural structure.

Given the relatively balanced Th1 and Th2 immunity [74] and the demonstrated potency of ALFQ in preclinical models [75], and CelTOS-induced cell-mediated immunity [36], we sought to characterize cellular immune responses induced by PfCel-PfCel. The frequencies of PfCelTOS-specific IFN-γ- or IL-4-producing splenocytes of the BALB/c mice were quantified by ELISpot. Similar to the induced humoral response, characterization of the cellular response revealed that ALFQ enhanced the production of PfCelTOS-specific IFN-γ and IL-4 across all doses compared to SAPN delivered without adjuvant, as well as rPfCelTOS (Figure 5c). Although the mean frequencies of IFN-γ- and IL-4-producing splenocytes increased with dose, they did not significantly differ. To further explore cytokine production by PfCel-PfCel at the higher doses (3 and 10 μg); PfCelTOS-specific cytokine concentrations were determined using the Meso Scale Discovery immunoassay. As previously observed, adjuvant was required to induce cytokine production; with the exception of Th1-skewed TNF-α and pro-inflammatory KC/GRO (Figure 5d). The elicited PfCelTOS-specific cytokine concentrations did not statistically differ between the low (3 μg) and the high dose (10 μg). Together, these results suggest that adjuvant was required to elicit potent humoral and cellular responses, and that increased epitope density through dual-display of PfCelTOS on PfCel-PfCel SAPN improved cellular, but not humoral, immunity relative to the soluble, rPfCelTOS.

## 4. Discussion

De novo, computationally designed self-assembling protein nanoparticles (SAPN) provide a modular strategy to present well-defined targets to the host immune system. This type of multi-valency offers the possibility to enhance immune responses through the simultaneous cross-linking of epitopes on nanoparticles and host B cell receptors, leading to efficient immune activation, thus overcoming the limitations of subunit or conventional vaccine approaches. A major advantage of the SAPN technology is the ability to include two B cell antigens, one on each terminus of the monomer. We demonstrated that two antigens expressed from different stages of the malaria life cycle could be displayed on the surface of a protein nanoparticle. We investigated the influence of the target antigen’s orientation on the SAPN monomer on immunity in mice and rabbits. Notably, immune responses were dependent on localization, protein disulfide-bridge structure, and epitope accessibility. Disulfide bond formation prior to urea denaturation and refolding of PfMSP1_19_ SAPN improved recognition by disulfide-dependent mAbs, suggesting some correct folding, and improved in vivo immune responses. Alternately, PfCelTOS displayed in various orientations yielded variable immune recognition that was likely more dependent on localization and epitope accessibility. Interestingly, PfCelTOS displayed from the C-terminal position more closely mimics the natural protein folding in this region, being primarily unconstrained. While for PfMSP1_19_, the opposite is true, where disulfide structure rather than positional location had a greater impact on immunogenicity given that correct disulfide structure at the N-terminus overcame the potentially less favorable localization on SAPN. Contrary to the soluble PfCelTOS, native MSP1_19_ is naturally constrained on the parasite membrane surface through a GPI anchor, thus the N-terminal localization is potentially more native-like in structure [17,19]. While these are compelling observations, they highlight the importance of protein folding and antigen localization for SAPN target display.

Proper folding of proteins with numerous disulfide bridges can be especially challenging. *E. coli* as an expression system has advantages as well as disadvantages. Notably, the cytosolic environment of *E. coli* is reductive, thus restricting the spontaneous formation of disulfide bridges. These bonds play a critical role in stabilizing protein structures, allowing for crosslinking between non-consecutive polypeptide segments. PfMSP1_19_ has a complex folded structure with six disulfide bridges that dictate its final conformation. The formation of correct disulfide bonds prior to urea denaturation appear to guide the subsequent refolding steps, confirmed by positive immunoreactivity on dot blots with MSP1_19_ conformation-dependent mAbs. The correctly folded PfMSP1_19_ on ^ox^PfMSP1_19_-PfCelTOS significantly improved immunogenicity in mice compared to the alternate purified form, ^red^PfMSP1_19_-PfCelTOS, leading to a ten-fold increase in geometric mean antibody concentrations. As such, formation of correct disulfide-dependent structures is essential to eliciting functional antibody responses against native malarial antigens, i.e., CSP, TRAP, AMA1, MSP1, Pfs25, Pfs48/45 [76,77,78], to name a few.

A number of studies have reported that antibodies to the C-terminal MSP1_42_ [16] and/or MSP1_19_ [79,80,81] are associated with protection in nonhuman primate and murine infection models, respectively, and that antibodies to MSP1_19_ are associated with reduced risk of symptomatic *P. falciparum* infection in the field [82]. To explore whether SAPN can induce in vitro functional antibodies as a surrogate to a potentially protective response, New Zealand white rabbits were vaccinated with SAPNs displaying PfMSP1_19_ in AddaVax^TM^. Contrary to the mouse model, examining PfMSP1_19_ immunogenicity in rabbits uncovered that N-terminal PfMSP1_19_ on SAPN was less immunogenic, resulting in lower PfMSP1 titers and concomitantly lower inhibition of parasite growth. The opposite was true for the PfCelTOS-^ox^PfMSP1_19_ SAPN, where localizing PfMSP1_19_ on the C-terminus induced significant inhibition that was comparable to rPfMSP1_42_. Differences in responses between mice and rabbits may be partially related to antigen processing and presentation [83]. The impact of epitope accessibility on SAPN and humoral responses was reported for another asexual blood-stage antigen, P27 [84]. Unlike for PfMSP1_19_, the surface exposed portions of the P27-NC SAPN, i.e., the C-terminal portion, induced a stronger immune response compared to responses against the N-terminal region, which was concealed within the core of the SAPN. Alternatively, others have reported the importance of T helper epitope regions on MSP1_33_ for the production of anti-MSP1_19_ inhibitory antibodies [85,86]. Thus, the inferior anti-MSP1_19_ antibody response in rabbits relative to rPfMSP1_42_ may be partially due to the lack of antigen-specific T-cell help from the MSP1_33_ portion of MSP1_42_. While our data reveal interesting trends, further investigation is needed to elucidate the mechanism of B-cell receptor recognition and differentiation of high-epitope-density nanoparticles.

For CelTOS, the C-terminal localization of PfCelTOS yielded improved immune recognition that was largely targeted to the C-terminus of the PfCelTOS protein. Immune responses targeting this portion of the molecule are biologically relevant, since genetic diversity and single nucleotide polymorphisms in the *P. falciparum celtos* gene are localized to this region [87]. Orienting the immunodominant C-terminal end on the C-terminus of the monomer of the assembled SAPN yielded a preferred, unconstrained and free-folding structure. Conversely, PfCelTOS displayed on the N-terminus yielded overall reduced immune responses, likely due to occlusion of the C-terminal end in the SAPN core. This configuration may conceal immunodominant epitopes or is evidence of inadequate display or antigen presentation. Improvements can be made to SAPN by either including longer linker segments between the N-terminus of the target and the core structure, or by displaying antigens in the reverse orientation. These findings reveal that for some target antigens, orientation on SAPN may be critical to elicit relevant responses.

The influence of CelTOS localization on immunogenicity was further explored by measuring cell-mediated immune responses elicited by a dual-antigen displaying PfCel-PfCel SAPN. While PfCel-PfCel did not necessarily improve humoral responses compared to a soluble rPfCelTOS, the PfCel-PfCel yielded higher numbers of cytokine-producing cells reactive to PfCelTOS compared to the soluble protein. This may be due to the repetitive nature, high-density display on SAPN activating cellular recruitment in conjunction with efficient delivery of increased molar equivalents by the two copies of PfCelTOS on PfCel-PfCel SAPN. Previously, we reported that mice immunized with rPfCelTOS/ISA 720 elicited CelTOS-specific antibodies, CD4^+^ and CD8^+^ T cells, and protection from heterologous infective challenge using *Plasmodium berghei (Pb)* sporozoites, demonstrating heterologous cross-epitope recognition or presentation [35]. BALB/c mice immunized with rPbCelTOS/ISA 720 yielded homologous protective immunity that was partially dependent on antibodies and CD4^+^ and CD8^+^ T cells to PbCelTOS [36]. Conversely, more recently, viral vectors encoding PfCelTOS did not yield protective immunity in either BALB/c or CD1 mice using rodent malaria transgenic parasites, even in the presence of antibodies and PfCelTOS-specific CD4+ and CD8+ T cells [88]. Collectively, while nominally identified as a conserved antigen and immunogenic in various mouse strains, CelTOS has yet to present clear evidence of sterile protection against *falciparum* species targets.

In summary, an ideal multi-antigen SAPN vaccine can be designed to present both B- and T-cell epitopes. These ordered antigenic arrays present high epitope densities that facilitate simultaneous binding between nanoparticles and host B cell receptors, leading to induction of potent immune responses. SAPN have been used to present peptides that form epitopes on the surface by the nature of their proximity and, depending on the target, variably elicit conformation-dependent neutralizing antibodies [62,63,64]. Importantly, SAPN can be applied to present structural B cell antigens, overcoming limitations of constrained genetic fusions of conformational antigens and their assembly on the surface of virus capsid proteins, i.e., VLPs [89,90,91]. Recently, a SAPN expressing segments of the malaria *P. falciparum* CSP, the C-terminal structural domain, α-TSR, on the N-terminus, and the central (NANP)_6_ repeat on the C-terminus was cGMP-produced and advanced to a first in human clinical investigation (FMP014/ALFQ; ClinicalTrials.gov Identifier: NCT04296279). An optimized PfCSP/PvCSP-based SAPN incorporating several different HLA and CSP-specific CD8+ T cell epitopes and the universal CD4+ T-helper Pan DR epitope (PADRE) induced protective cellular immune responses in mice, demonstrating the versatility of the platform [92]. Here, bivalent SAPN were used to elicit humoral and cellular immune responses in vivo. Relative to orientation on the SAPN, immune responses were dependent on the antigen localization, epitope accessibility, and on the disulfide bond structure for MSP1. These findings suggest that a characteristic understanding of the SAPN platform and the antigen target is essential to develop a multi-antigen, combination vaccine using this approach. Taken together, these results demonstrate that the SAPN approach is sufficiently adaptable for multiple structural antigens display.

## 5. Conclusions

The present study provides evidence for the influence of antigen localization and protein folding, as well as epitope density on SAPN on the host immune response. The immunogenicity of SAPN-presenting malaria antigens, PfCelTOS and PfMSP1_19_, in various orientations was characterized in mice and rabbits. SAPN displaying PfCelTOS on the C-terminus achieved superior PfCelTOS-specific antibody concentrations compared to N-terminal localization, which occluded important epitopes in the SAPN core. The formation of proper disulfide bonds in PfMSP1_19_ by an oxidative purification approach, confirmed by immunoreactivity to conformation-dependent monoclonal antibodies, yielded increased immunogenicity relative to PfMSP1_19_-displaying SAPN purified by a fully denaturing and reductive process. Importantly, PfMSP1_19_-specific antibodies were functional against blood-stage parasite growth. Increased epitope density through display of PfCelTOS on both termini of SAPN yielded improved cellular, but not humoral, responses. This work highlights the potential advantages of using SAPN as scaffolds for multivalent antigen display. A systematic investigation of the structural determinants and their influence on immunogenicity highlights the importance of antigen selection and design. Thus, SAPN lends itself well to a de novo computational design approach for vaccine development.

## 6. Patents

Peter Burkhard holds intellectual property on the use of SAPN platform as a vaccine technology. Zoltan Beck is an inventor in a US-government-owned and issued US patent based on ALFQ adjuvant. Evelina Angov and Elke Bergmann-Leitner are inventors in a US-government-owned and issued US patent based on the recombinant soluble PfCelTOS. Evelina Angov is an inventor in US-government owned and US patent based on recombinant, soluble PfMSP1_42_ (FVO).

## Data Availability

The datasets generated and/or analyzed during the current study are available from the corresponding author on reasonable request.

## Supplementary Materials

The following are available online at https://www.mdpi.com/2076-393X/9/2/103/s1, Figure S1: SAPN amino acid sequences. Figure S2: Rabbit anti-PfCelTOS and purified IgG PfMSP1_19_ responses, Figure S3: Reactivity of polyclonal anti-PfCel-PfCel and anti-rPfCelTOS antibodies against *P. falciparum* sporozoites. Table S1: Purification buffer conditions by column for the SAPN, Table S2: Buffer conditions for stepwise refolding of the SAPN.
