# Supplementary material for: Orientation of Antigen Display on Self-Assembling Protein Nanoparticles Influences Immunogenicity
Source: Vaccines (Basel). 2021 Jan 29;9(2):103. doi: 10.3390/vaccines9020103 (PMC7911071; doi:10.3390/vaccines9020103)
Supplement: Supplementary file 1 [file vaccines-09-00103-s001.pdf]

Supplementary Figure 1. SAPN amino acid sequences

PfMSP1$_{19}$-PfCelTOS (360 aa, ~39.6KDa)

MGHHHHHHRNISQHQCVKKQCPQNSGCFRHLDEREECKCLLNYKQEGDKCVENPNPTCNENNGGCDADAKCTEED
SGSNGKKITCECTKPDSYPLFDGIFCSASNRGSGSWERWNAKWDEWRNDQNDWREDWQAWRDDWAYWTLTWR
YGELYSRLARIERRVEELRRLLQLIRHENRMVLQFVRALSMQARRLEKGRSARGTFRGNNGHNSSSSLYNGSQFIEQLN
NSFTSAFLESQSMNKIGDDLAETISNELVSVLQKNSPTFLESSFDIKSEVKKHAKSMLKELIKVGLPSFENLVAENVKPPKV
DPATYGIIVPVLTSLFNKVETAVGAKVSDEIWNYNSPDVSESEESLSDDFFD

PfCelTOS-PfMSP1$_{19}$ (360 aa, ~39.6KDa)

MGHHHHHHRTFRGNNGHNSSSSLYNGSQFIEQLNNSFTSAFLESQSMNKIGDDLAETISNELVSVLQKNSPTFLESSFDI
KSEVKKHAKSMLKELIKVGLPSFENLVAENVKPPKVDPATYGIIVPVLTSLFNKVETAVGAKVSDEIWNYNSPDVSESEES
LSDDFFDASNRGSGSWERWNAKWDEWRNDQNDWREDWQAWRDDWAYWTLTWRYGELYSRLARIERRVEELRR
LLQLIRHENRMVLQFVRALSMQARRLEKGRSARGNISQHQCVKKQCPQNSGCFRHLDEREECKCLLNYKQEGDKCVE
NPNPTCNENNGGCDADAKCTEEDSGSNGKKITCECTKPDSYPLFDGIFCS

PfCelTOS-PfCelTOS (425aa, ~46.75kDa)

MGHHHHHHRFRGNNGHNSSSSLYNGSQFIEQLNNSFTSAFLESQSMNKIGDDLAETISNELVSVLQKNSPTFLESSFDI
KSEVKKHAKSMLKELIKVGLPSFENLVAENVKPPKVDPATYGIIVPVLTSLFNKVETAVGAKVSDEIWNYNSPDVSESEES
LSDDFFDASNRGSGSWERWNAKWDEWRNDQNDWREDWQAWRDDWAYWTLTWRYGELYSRLARIERRVEELRR
LLQLIRHENRMVLQFVRALSMQARRLEKGRSARGTFRGNNGHNSSSSLYNGSQFIEQLNNSFTSAFLESQSMNKIGDD
LAETISNELVSVLQKNSPTFLESSFDIKSEVKKHAKSMLKELIKVGLPSFENLVAENVKPPKVDPATYGIIVPVLTSLFNKVET
AVGAKVSDEIWNYNSPDVSESEESLSDDFFD

Supplementary Table 1. Purification buffer conditions by column for the SAPN.

Buffer	Component	^{red}PfMSP1$_{19}$-PfCelTOS	^{ox}PfMSP1$_{19}$-PfCelTOS	PfCelTOS-^{ox}PfMSP1$_{19}$	PfCelTOS-PfCelTOS
**Ni-NTA**					
Lysis Buffer(pH 8.0)	Monobasic Sodium Phosphate	*20mMTris-HCl 6MGuanidine-HCl*	20mM	20mM	20mM
	SodiumChloride		50mM	50mM	50mM
Equilibration (pH 8.0)	Monobasic Sodium Phosphate	*20mMTris-HCl 5mMTCEP-HCl 8MUrea*	20mM	20mM	20mM
	Sodium Chloride		500mM	500mM	1M
	Sarcosyl		0.3%	0.3%	0.3%
	Imidazole		N/A	N/A	7mM
Wash I (pH 8.0)	Monobasic Sodium Phosphate	*20mMTris-HCl 5mMTCEP-HCl 450mMSodiumChloride 8MUrea*	20mM	20mM	20mM
	Sodium Chloride		50mM	50mM	50mM
	Sarcosyl		0.1%	0.1%	0.1%
	Imidazole		N/A	N/A	7mM
Wash II (pH 8.0)	Tris-HCl	20mM	N/A	N/A	N/A
	TCEP-HCl	5mM			
	Imidazole	25mM			
	Urea	8M			
Elution (pH 8.0)	Monobasic Sodium Phosphate	*10mMSodiumCitrate 50mMSodiumChloride 250mMImidazole 8MUrea (pH6.0)*	20mM	20mM	20mM
	Sodium Chloride		50mM	50mM	50mM
	Imidazole		250mM	250mM	250mM
Imidazole		1M	1M	1M	1M
**SP-Sepharose**					
Equilibration (pH 6.0)	Urea	8M	8M	8M	N/A
	Sodium Citrate	10mM	10mM	10mM	
	Sodium Chloride	50mM	50mM	50mM	
Wash (pH 6.0)	Urea	8M	8M	8M	
	Sodium Citrate	10mM	10mM	10mM	
	Sodium Chloride	100mM	120mM	100mM	
Elution (pH 8.0)	Urea	8M	8M	8M	
	Tris-HCl	10mM	10mM	10mM	
	Sodium Chloride	450mM	450mM	450mM	
**Q-Sepharose**					
Equilibration (pH 8.0)	Urea	N/A	8M	8M	8M
	Tris-HCl		10mM	10mM	10mM
	Sodium Chloride		50mM	50mM	50mM
Wash (pH 8.0)	Urea		8M	8M	8M
	Tris-HCl		10mM	10mM	10mM
	Sodium Chloride		150mM	100mM	120mM
Elution (pH 8.0)	Urea		8M	8M	8M
	Tris-HCl		10mM	10mM	10mM
	Sodium Chloride		300mM	250mM	350mM
	Arginine		N/A	N/A	100mM

Supplementary Table 2. Buffer conditions for stepwise refolding of the SAPN.

Buffer	Component	^{red}PfMSP1$_{19}$-PfCelTOS	^{ox}PfMSP1$_{19}$-PfCelTOS	PfCelTOS-^{ox}PfMSP1$_{19}$	PfCelTOS-PfCelTOS
Refolding Buffer 1	pH	7.5	7.5	7.5	8.5
	**Urea**	**8M**	**8M**	**8M**	**8M**
	Tris-HCl	40mM	40mM	40mM	40mM
	Sodium Chloride	150mM	150mM	150mM	40mM
	Sucrose	N/A	10%	N/A	N/A
	Glycerol	5%	5%	5%	5%
	TCEP-HCl	5mM	N/A	5mM	N/A
	Arginine	N/A	N/A	N/A	500mM
Refolding Buffer 2	pH	7.5	7.5	7.5	8.5
	**Urea**	**6M**	**6M**	**6M**	**6M**
	Tris-HCl	40mM	40mM	40mM	40mM
	Sodium Chloride	150mM	150mM	150mM	40mM
	Sucrose	N/A	10%	N/A	N/A
	Glycerol	5%	5%	5%	5%
	TCEP-HCl	5mM	N/A	5mM	N/A
	Arginine	N/A	N/A	N/A	400mM
Refolding Buffer 3	pH	7.5	7.5	7.5	8.5
	**Urea**	**4M**	**4M**	**4M**	**4M**
	Tris-HCl	40mM	40mM	40mM	40mM
	Sodium Chloride	150mM	150mM	150mM	40mM
	Sucrose	N/A	10%	N/A	N/A
	Glycerol	5%	5%	5%	5%
	TCEP-HCl	5mM	N/A	5mM	N/A
	Arginine	N/A	N/A	N/A	300mM
Refolding Buffer 4	pH	7.5	7.5	7.5	8.5
	**Urea**	**2M**	**2M**	**2M**	**2M**
	Tris-HCl	40mM	40mM	40mM	40mM
	Sodium Chloride	150mM	150mM	150mM	40mM
	Sucrose	N/A	10%	N/A	N/A
	Glycerol	5%	5%	5%	5%
	TCEP-HCl	N/A	N/A	N/A	N/A
	Arginine	N/A	N/A	N/A	200mM
Refolding Buffer 5	pH	7.5	7.5	7.5	8.5
	Tris-HCl	40mM	40mM	40mM	40mM
	Sodium Chloride	150mM	150mM	150mM	40mM
	Sucrose	N/A	10%	N/A	N/A
	Glycerol	5%	5%	5%	5%
	TCEP-HCl	N/A	N/A	N/A	N/A
	Arginine	N/A	N/A	N/A	N/A

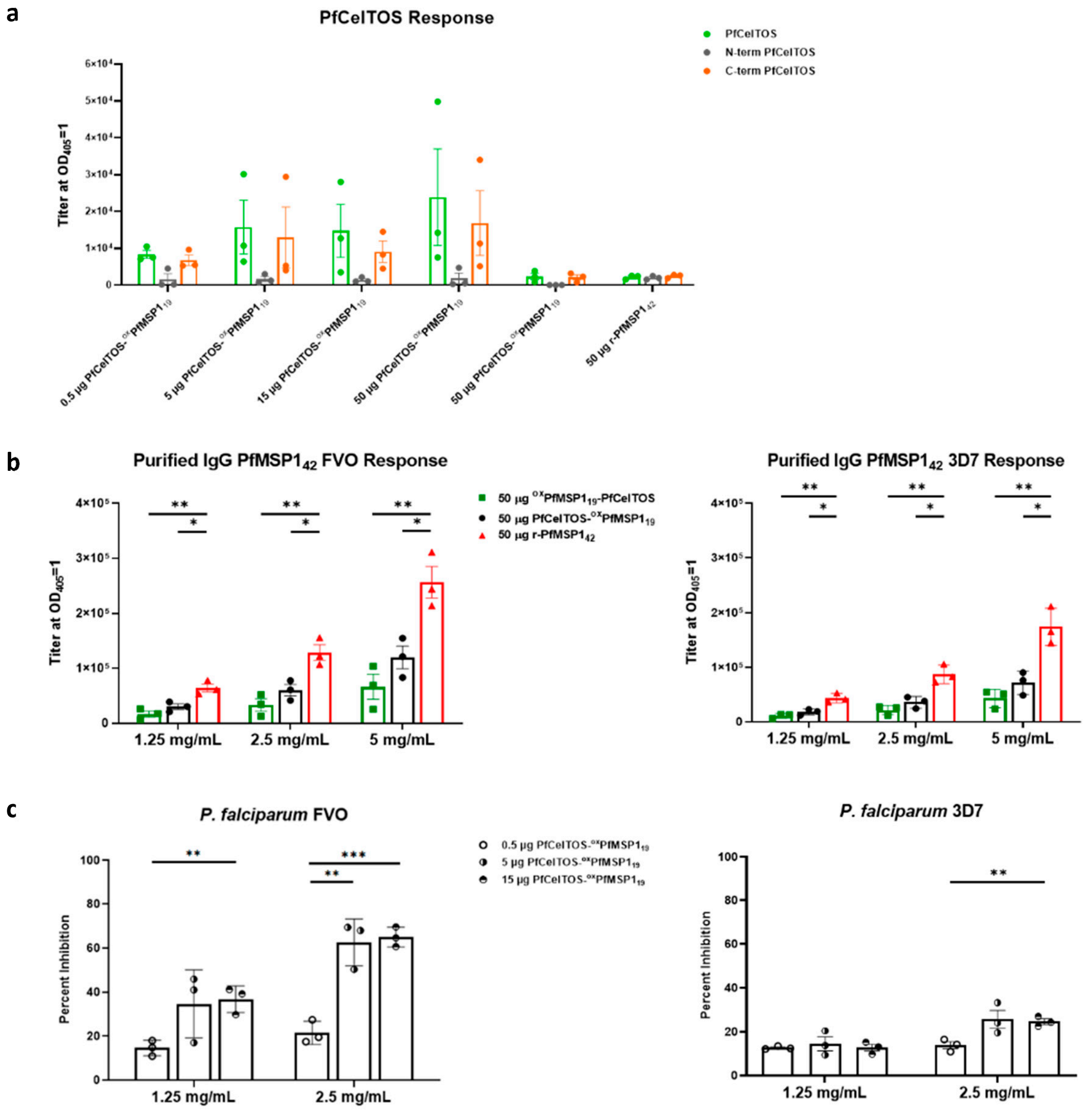

Supplementary Figure 2. Rabbit anti-PfCelTOS and purified IgG PfMSP1$_{19}$ responses. (**a**) Rabbit sera ELISA titers for antigen-specific antibodies to the full length, N-term, and C-term of PfCelTOS. (**b**) Purified IgG from rabbits immunized with 50 µg of SAPN or recombinant protein measuring FVO and 3D7 PfMSP1$_{42}$-specific antibodies at 1.25, 2.5, and 5 mg/mL. (**c**) PfCelTOS-^{ox}PfMSP1$_{19}$ purified IgG dose titration measuring growth inhibition of the FVO and 3D7 parasite strains at 1.25 and 2.5 mg/mL. Data are shown as the mean with +/- standard error

of the mean and were analyzed for statistical difference using nonparametric multiple t tests, Holm-Sidak method.

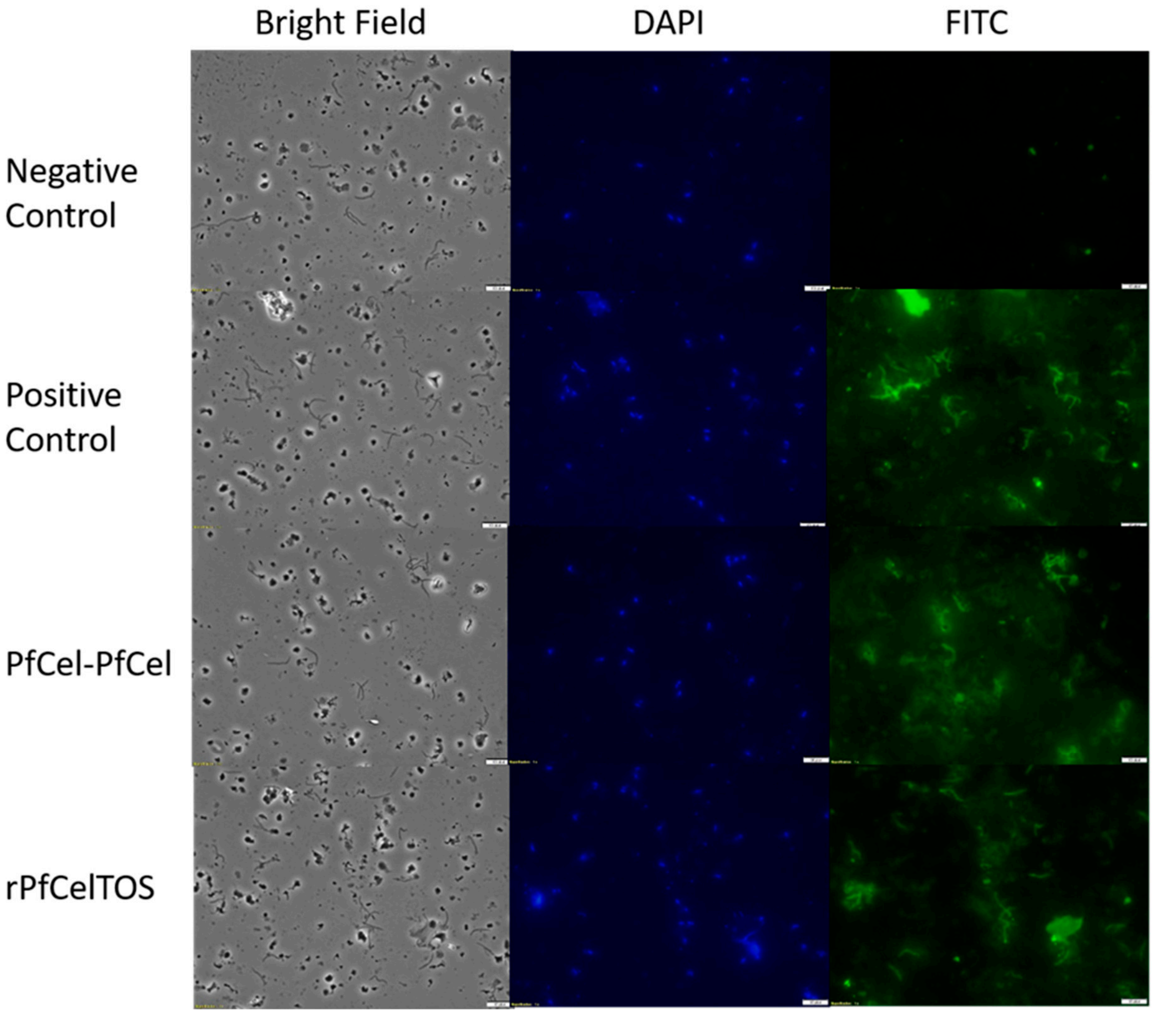

Supplementary Figure 3. Reactivity of polyclonal anti-PfCel-PfCel and anti-rPfCelTOS antibodies against *P. falciparum* sporozoites. The antiserum used for the staining was collected two weeks following the third immunization and was tested at 1:200 dilution. Pooled pre-immunization serum and pooled anti-PfCelTOS serum from BALB/c mice immunized with recombinant PfCelTOS in Montanide were used for negative and positive controls, respectively. Images were taken at 1,000x magnification.
